# Supplementary material for: Modeling glioblastoma heterogeneity as a dynamic network of cell states
Source: Mol Syst Biol. 2021 Sep 16;17(9):e10105. doi: 10.15252/msb.202010105 (PMC8444284; doi:10.15252/msb.202010105)
Supplement: Supplementary file 6 — Source Data for Figure 5 [file MSB-17-e10105-s004.zip › Figure5A_sourcedata/GSEA_3071/nosigdb_stateIII.GseaPreranked.1622795155562/OLSEN_SCPLIKE.html]

Details for gene set OLSEN\_SCPLIKE[GSEA]

|  || Dataset | state33071 |
| Phenotype | NoPhenotypeAvailable |
| Upregulated in class | na\_neg |
| GeneSet | OLSEN\_SCPLIKE |
| Enrichment Score (ES) | -0.63770336 |
| Normalized Enrichment Score (NES) | -3.4061296 |
| Nominal p-value | 0.0 |
| FDR q-value | 0.0 |
| FWER p-Value | 0.0 |
Table: GSEA Results Summary

  

Fig 1: Enrichment plot: OLSEN\_SCPLIKE      
 Profile of the Running ES Score & Positions of GeneSet Members on the Rank Ordered List

  

| PROBE | GENE SYMBOL | GENE\_TITLE | RANK IN GENE LIST | RANK METRIC SCORE | RUNNING ES | CORE ENRICHMENT || 1 | MT2A |  |  | 31 | 0.499 | 0.0082 | No |
| 2 | S100A11 |  |  | 48 | 0.464 | 0.0191 | No |
| 3 | RHOC |  |  | 92 | 0.414 | 0.0214 | No |
| 4 | S100A10 |  |  | 102 | 0.409 | 0.0323 | No |
| 5 | S100A6 |  |  | 172 | 0.360 | 0.0262 | No |
| 6 | LGALS1 |  |  | 230 | 0.323 | 0.0219 | No |
| 7 | TOMM7 |  |  | 264 | 0.303 | 0.0232 | No |
| 8 | MYL9 |  |  | 293 | 0.293 | 0.0255 | No |
| 9 | NTM |  |  | 553 | -0.257 | -0.0329 | No |
| 10 | OLFML2A |  |  | 635 | -0.261 | -0.0453 | No |
| 11 | PMP22 |  |  | 1023 | -0.281 | -0.1360 | No |
| 12 | COL18A1 |  |  | 1302 | -0.295 | -0.1981 | No |
| 13 | PRNP |  |  | 1309 | -0.295 | -0.1901 | No |
| 14 | TIMP1 |  |  | 2038 | -0.337 | -0.3668 | No |
| 15 | CTSD |  |  | 2110 | -0.342 | -0.3740 | No |
| 16 | ZEB2 |  |  | 2484 | -0.372 | -0.4581 | No |
| 17 | SPTBN1 |  |  | 2647 | -0.385 | -0.4874 | No |
| 18 | PSAP |  |  | 2808 | -0.400 | -0.5157 | No |
| 19 | ITGA6 |  |  | 2899 | -0.408 | -0.5257 | No |
| 20 | ITGB8 |  |  | 3026 | -0.424 | -0.5444 | No |
| 21 | CD63 |  |  | 3258 | -0.452 | -0.5893 | No |
| 22 | ITM2B |  |  | 3314 | -0.463 | -0.5885 | No |
| 23 | ZFP36L1 |  |  | 3487 | -0.503 | -0.6166 | No |
| 24 | VMP1 |  |  | 3570 | -0.536 | -0.6204 | Yes |
| 25 | ITGB1 |  |  | 3574 | -0.537 | -0.6038 | Yes |
| 26 | DST |  |  | 3597 | -0.545 | -0.5918 | Yes |
| 27 | PLAT |  |  | 3600 | -0.547 | -0.5746 | Yes |
| 28 | IGFBP7 |  |  | 3611 | -0.554 | -0.5592 | Yes |
| 29 | ERBB3 |  |  | 3617 | -0.558 | -0.5424 | Yes |
| 30 | CD9 |  |  | 3634 | -0.567 | -0.5282 | Yes |
| 31 | KCTD12 |  |  | 3646 | -0.574 | -0.5124 | Yes |
| 32 | COL4A2 |  |  | 3664 | -0.586 | -0.4978 | Yes |
| 33 | PMEPA1 |  |  | 3737 | -0.657 | -0.4952 | Yes |
| 34 | TIMP3 |  |  | 3755 | -0.677 | -0.4776 | Yes |
| 35 | LIMA1 |  |  | 3774 | -0.706 | -0.4594 | Yes |
| 36 | APP |  |  | 3779 | -0.710 | -0.4374 | Yes |
| 37 | SPARC |  |  | 3811 | -0.759 | -0.4209 | Yes |
| 38 | COL4A1 |  |  | 3829 | -0.791 | -0.3996 | Yes |
| 39 | FN1 |  |  | 3850 | -0.842 | -0.3775 | Yes |
| 40 | S100B |  |  | 3853 | -0.845 | -0.3507 | Yes |
| 41 | GAS7 |  |  | 3871 | -0.933 | -0.3249 | Yes |
| 42 | PMP2 |  |  | 3876 | -0.959 | -0.2949 | Yes |
| 43 | NEAT1 |  |  | 3880 | -1.005 | -0.2631 | Yes |
| 44 | GPM6B |  |  | 3889 | -1.047 | -0.2313 | Yes |
| 45 | APOE |  |  | 3891 | -1.056 | -0.1974 | Yes |
| 46 | CRYAB |  |  | 3894 | -1.078 | -0.1630 | Yes |
| 47 | IGFBP5 |  |  | 3895 | -1.082 | -0.1280 | Yes |
| 48 | COL1A2 |  |  | 3906 | -1.278 | -0.0892 | Yes |
| 49 | PTPRZ1 |  |  | 3913 | -1.342 | -0.0474 | Yes |
| 50 | MALAT1 |  |  | 3924 | -1.583 | 0.0013 | Yes |
Table: GSEA details [plain text format]

  

Fig 2: OLSEN\_SCPLIKE: Random ES distribution      
 Gene set null distribution of ES for **OLSEN\_SCPLIKE**
